# The protective role of curcumin in mitigating drug-induced toxicity in male reproductive system

**DOI:** 10.3389/fphar.2025.1620732

**Published:** 2025-07-28

**Authors:** Mitra Tarlan, Shabnam Moradi, Niloofar Heidarizade, Omid Tavallaei, Saeed Khazayel, Mohamad Hosein Farzaei, Javier Echeverría

**Affiliations:** ^1^Pharmaceutical Sciences Research Center, Health Institute, Kermanshah University of Medical Sciences, Kermanshah, Iran; ^2^Departamento de Ciencias del Ambiente, Facultad de Química y Biología, Universidad de Santiago de Chile, Santiago, Chile

**Keywords:** curcumin, male reproductive toxicity, oxidative stress, drug-induced toxicity, antioxidants

## Abstract

**Background:**

Curcumin, a key bioactive component of turmeric (*Curcuma longa* L. [Zingiberaceae]), has gained considerable attention for its potential to mitigate drug-induced toxicity. This review synthesizes and clarifies current findings on curcumin’s ability to prevent the adverse effects of various pharmaceuticals.

**Methods:**

A comprehensive search using multiple databases—PubMed^®^, Scopus^®^, ScienceDirect^®^, and Web of Science®—was conducted for articles published up to October 2023. The current review is limited to randomized controlled trials, observational studies, and animal studies investigating the protective role of curcumin against drug-induced toxicity. The data extraction process included a variety of study characteristics, types of drugs used, curcumin dosing regimens, and reported outcomes associated with drug-induced toxicity.

**Results:**

A total of twenty-five studies were reviewed for this analysis. Curcumin may help reduce the side effects of certain medications, including sertraline, diclofenac, paclitaxel, irinotecan, and methotrexate.

**Discussion:**

Research also indicates that curcumin possesses antioxidant properties, reduces inflammation, and aids sperm production. Most importantly, sperm motility, density, and morphology significantly improved in curcumin-treated groups compared to the control groups undergoing toxic pharmaceutical treatment. The dosage of curcumin used in these studies ranges from 50 to 200 mg/kg body weight.

**Conclusion:**

The available evidence suggests that curcumin may serve as a protective agent for male reproductive health against drug-induced damage, based on its diverse effects in mitigating oxidative stress and inflammation, which provide potential use in preserving reproductive health in males during pharmacological interventions. However, standardization of methodologies, along with more clinical evidence, is highly required before the practical application of findings related to treatment benefits can be made. Subsequent studies should focus on optimizing the use of this compound in combination with other pharmacological agents to enhance the protective effects of curcumin on male reproductive health.

## 1 Introduction

### 1.1 Definition, epidemiology, and risk factors of male infertility

Male infertility, which is mainly caused by sperm ejaculation, oligozoospermia (low sperm count), poor sperm motility, and morphological variations in the sperm, has only recently received clinical and scientific attention (Agarwal et al., 2021). According to epidemiological reports, male factors account for around 20%–30% of infertility, or 50% of cases overall (Wei et al., 2017). Surprisingly, idiopathic infertility still accounts for 30%–40% of male infertility (Halder, 2017; Katz et al., 2017). A growing body of research has shown that several factors, containing physiological ones (age, weight, body composition) (Keszthelyi et al., 2020), pathological ones (metabolic disorders, inflammation) (Morrison and Brannigan, 2015; Agarwal et al., 2018), psychological ones (trauma and stress)(Joja et al., 2015), lifestyle ones (smoking, alcohol consumption, and drug abuse) (Durairajanayagam, 2018), and environmental ones (exposure to toxins and heavy metals) (Ilieva et al., 2020), can affect sexual fertility in men. Oxidative stress, inflammation, and apoptosis are frequent processes in the prognosis of male infertility and provide a diagnostic answer for the majority of instances of idiopathic male infertility (Ojo et al., 2023). All the factors discussed here and beyond can influence these mechanisms.

### 1.2 Oxidative stress is one of the mechanisms involved in male infertility

Oxidative stress is one of the most threatening factors and a decisive factor for male infertility; it affects sperm function and inhibits germ cell development. Oxidative stress is a condition in which the level of reactive oxygen species (ROS) produced exceeds the cell’s endogenous antioxidant defense system. Protein, lipid, deoxyribonucleic acid (DNA), and ribonucleic acid (RNA) – all the biological macromolecules–can be affected by ROS (Samavarchi Tehrani et al., 2019). ROS, which can be produced by internal processes or in reaction to external stimuli, is prevented from harming cells by the endogenous cellular antioxidant defense mechanism (Aitken et al., 2022). These ROS activities can reduce normal sperm parameters, including motility, count, and viability, as well as the ability to fertilize the oocyte, and damage the sperm head and tail structure, as well as sperm DNA (Alahmar, 2019; Martins et al., 2021). Due to the synthesis of large amounts of ROS in the testis, spermatogenesis and steroidogenesis are highly sensitive to damage, even when the intratesticular blood supply is inadequate and oxygen concentration is low (Aitken and Roman, 2009). Moreover, it is well-established that elevated ROS levels contribute to inflammation, which can be caused by various diseases such as obesity, smoking, alcoholism, and varicocele (Agarwal et al., 2018). Furthermore, the testis appears to be very sensitive to exogenous factors, for example, various therapeutic drugs that can potentially affect spermatogenic capacity as well as sperm quality and sexual activity (Ajayi and Akhigbe, 2020). Therefore, the improvement of oxidative stress through the use of antioxidant compounds, which reduce ROS, can help mitigate damage to testis tissue and improve sperm parameters.

### 1.3 Inflammation is one of the mechanisms involved in male infertility

In addition, male reproductive tract inflammation creates vicious loops that affect male reproductive tissues both physically and functionally, and it is tightly linked to oxidative stress (Dutta et al., 2020). Ample evidence has indicated that inflammation is correlated to diseases of the reproductive system, which impact men’s fertility (Azenabor et al., 2015; Hasan et al., 2022; Fomichova et al., 2024). The male reproductive system can become inflamed for several reasons, such as inflammation of the epididymis, blockage of the ejaculatory duct, and sexually transmitted infections such as *Chlamydia*, *Escherichia coli*, and gonorrhea (Azenabor et al., 2015; Frungieri et al., 2018). Essential questions in modern medicine relate to the direct correlation between the onset of infertility and acute or chronic inflammation. It was elucidated that dysregulation of spermatogenesis, restriction of sperm transport, and impairment of accessory gland activities can all contribute to the worse quality of semen during the inflammatory process (Ojo et al., 2023). In addition to the natural oxidative stress produced by sperm cells, the inflammatory process generates ROS, which have detrimental effects on human spermatozoa (Azenabor et al., 2015). Thereby, male infertility is caused by inflammation-causing testicular damage and infections. Hence, modulating oxidative stress and inflammation can be a promising therapeutic strategy for managing and treating male infertility.

### 1.4 Apoptosis is one of the mechanisms involved in male infertility

It is essential to comprehend certain aspects of apoptosis, a biological process inherent to living organisms, which involves events leading to a change in cell morphology and ultimately, cell death. A variety of internal and external factors that cause minor damage to the cell lead to apoptosis, which is then eliminated under supervision (Stringer et al., 2023). Somatic cell shrinkage, nuclear DNA fragmentation, chromatin condensation, dynamic membrane blebbing, and loss of adhesion to the extracellular matrix are hallmarks of the apoptotic pathway (Jan and Chaudhry, 2019).

In summary, idiopathic male infertility is attributed to a widespread increase in germ cell apoptosis under certain clinical conditions (Pareek et al., 2007). Spermatogenesis in the seminiferous epithelium is followed by germ cell apoptosis, a process that happens throughout life. It has been demonstrated that aberrant germ cells must undergo apoptosis to be removed and maintain the standard germ cell-to-Sertoli cell ratio, primarily during maturation (Evenson, 1999). Apoptosis plays a crucial role in preserving sperm quality by eliminating defective germ cells; however, excessive or dysregulated apoptosis—often induced by oxidative stress, inflammation, or environmental toxins—can lead to reduced sperm count, motility, and DNA integrity. In addition to apoptosis, other molecular pathways such as impaired autophagy, oxidative stress (*via* excessive ROS production), mitochondrial damage, and endoplasmic reticulum stress also contribute to reduced sperm quality (Wang et al., 2003; Sakkas and Alvarez, 2010; Ali and Ibrahim, 2018). Thus, it is especially crucial to regulate the rate of apoptosis in males to maintain fertility.

### 1.5 Curcumin’s significance in male infertility and the limitations of existing treatments

Infertility is an area where conventional treatment options, such as chemotherapy and surgery, can provide maximum intervention. However, they can sometimes be expensive, invasive, and ineffective, and can have several adverse effects (Jiang et al., 2017). Thus, finding novel and effective treatment drugs to treat infertility issues appears to be crucial. Traditional medicine has employed herbal plants and other medications to treat a wide range of reproductive health issues over the past decade. These medications exhibit significant biological benefits over conventional chemical pharmaceuticals, including reduced toxicity and side effects (Mohammadi et al., 2013). Among the numerous health benefits of natural polyphenols derived from medicinal plants is their ability to reduce inflammation and oxidative stress, which can aid in the treatment of infertility (Seddiki et al., 2017).

Curcumin, (1*E*,6*E*)-1,7-bis(4-hydroxy-3-methoxyphenyl)-1,6-heptadiene-3,5-dione, is known as one of the most popular polyphenols from turmeric. Turmeric is a yellow spice obtained from the rhizome of the plant *Curcuma longa* Linn [Zingiberaceae]. The plant is prepared by converting the extracted roots into a fine powder. Curcumin has three active functional groups: two phenolic groups and a diketone moiety. It is also found in other flavonoids such as procyanidins (Nemzer et al., 2019; Priyadarsini et al., 2020). Curcumin also undergoes several biological and chemical transformations, including hydrolysis, degradation, reversible and irreversible *N*-attack reactions, and hydrogen donation reactions, which can lead to the oxidation of curcumin (Priyadarsini et al., 2020). Curcumin has demonstrated several therapeutic effects due to its proven antioxidant effects. It is a potent scavenger of ROS, including nitrogen dioxide radicals, superoxide anion radicals, and hydroxyl radicals. Furthermore, it has been shown to inhibit lipid peroxidation in many animal models (Ak and Gülçin, 2008; Saifi et al., 2022). However, the effects of curcumin on various components of male fertility are unclear. Several studies have emphasized the role of curcumin in spermatogenesis, the maintenance of oxidative homeostasis in sperm cells, and its energizing and protective effects on testicular tissue (Soleimanzadeh and Saberivand, 2013; Tsao et al., 2022). Several authors have studied the effects of curcumin on male sex hormones, semen parameters, and testicular cells (Kalender et al., 2019; Chaithra and Sarjan, 2020). For example, they noted that after curcumin administration, total antioxidant capacity, malondialdehyde (MDA), C-reactive protein (CRP), and tumor necrosis factor-alpha (TNF-α) were significantly reduced in addition to total sperm count, concentration, and motility (Alizadeh et al., 2018). Additionally, this compound reduces the effects of toxic agents on sperm morphology while also increasing sperm concentration and motility. On the other hand, the results showed that testosterone concentration in the curcumin-treated group increased significantly compared to the fluoride and sodium fluoride-exposed groups. It also has a positive effect on modified spermatogenesis and histopathological changes in the testis (Chaithra and Sarjan, 2020). However, some reports suggest that curcumin is toxic at high concentrations to sperm quality, motility, and viability, and therefore, the toxicity of curcumin and its contraceptive effects require careful and thorough study. Consequently, it appears that this compound is bivalent, thus requiring further discussion regarding its impact on testicular cells, sperm production, and male hormonal profile, which will be explained in the following sections of this study (Naz, 2011; Xia et al., 2012; 2020; Mohebbati et al., 2017).

Recently, investigations on male reproductive health have reported on the effects of drugs, including toxicity. Co-administration of 100–400 mg/kg of curcumin, for instance, significantly restored testicular antioxidant enzymes such as superoxide dismutase (SOD) and glutathione peroxidase (GPx), decreased lipid peroxidation (MDA), and enhanced sperm count, motility, viability, and testicular histology in Wistar rats exposed to 50 mg/kg of lead acetate for 35 days (Sudjarwo et al., 2017).

Such toxic effects may include influences that affect sperm count and motility, hormones, and the shape and function of the male reproductive organs. The male reproductive system appears to be more sensitive to oxidative stress and inflammation that the use of certain drugs can induce. This sensitivity motivates the search for preventive agents that can prevent or minimize such conditions. The complex biological effects of curcumin may make this compound a protector against chemical-induced reproductive toxicity. Curcumin’s capacity to scavenge free radicals and modulate cytokine development for inflammatory processes suggests that this spice may protect male fertility. Several experimental studies in animals have shown that curcumin helps reduce the toxic effects of various chemicals on male fertility. These studies suggest that curcumin may enhance sperm quality and mitigate hormonal imbalances through its pharmacological effects (Moutabian et al., 2022). Furthermore, by regulating apoptosis and survival signals, curcumin may be considered a protective agent. Therefore, curcumin may enhance testicular function and promote general fertility by activating these pathways (Ombredane et al., 2021).

However, there is a need for systematic research synthesis on the protective role of curcumin against drug toxicity in the male reproductive system. This study also helps to clarify dosages, modes of action, and potential therapeutic applications. The present systematic review aims to analyze the literature on the protective effect of curcumin against drug-induced toxicities in the male reproductive system. In combining the results of multiple studies, we aim to highlight the positive effects of curcumin and analyze the inherent weaknesses in the existing research. Therefore, the conclusions of this review may have important clinical and public health implications for individuals vulnerable to toxic elements in drugs. Additionally, recommendations for future research avenues will be provided to inform further studies in this promising line of inquiry.

## 2 Materials and methods

### 2.1 Search strategy

This systematic review was conducted in accordance with the guidelines outlined in the PRISMA checklist. The scientific databases used in the current study were PubMed^®^, Scopus^®^, Web of Science^®^, and Google Scholar^®^, which were searched up to October 2023. The keywords and MeSH terms included in the search were for curcumin and the male reproductive system. The search string employed was: (male infertility OR male subfertility OR male sterility OR aspermia OR azoospermia OR sperm OR testicular tissue OR sex hormones OR semen OR oligozoospermia AND (curcumin OR nano curcumin)).

### 2.2 Inclusion and exclusion criteria

The review included studies that met the following criteria: i) Studies (*in vivo*, *in vitro*, or clinical) investigating the potential of curcumin to protect against drug toxicity in the male reproductive tract, ii) English-language journals. The use of journals was based on articles written in English, and iii) For this study, only articles and reviews published up to October 2023 were considered. The exclusion criteria were:i) Journals that excluded articles related to the male reproductive tract, ii) Only systematic reviews, meta-analyses, and editorial articles were included, iii) Studies in which no quantitative data on the effects of curcumin were used, iv) Experimental studies in which animals other than mammalian species were used, and v) Articles not written in English were excluded.

### 2.3 Data extraction

Most data extraction was performed using a standardized form. The data collected included information on the type of studies conducted, the drugs used, the doses of curcumin administered, and any reported effects related to drug toxicity.

## 3 Results

### 3.1 The role of curcumin in mitigating drug-induced reproductive toxicity in males

**FIGURE 1:**
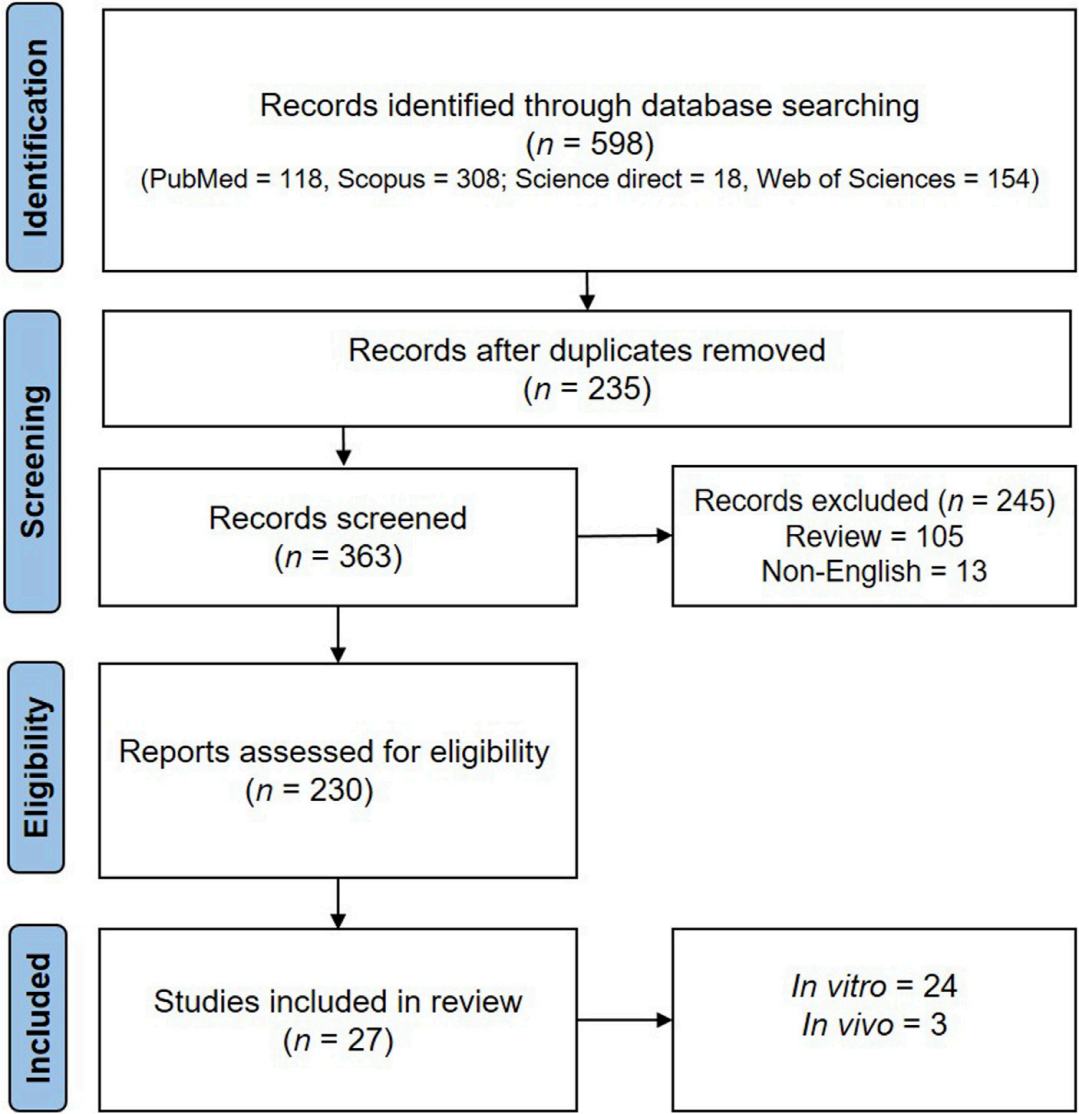
The PRISMA flowchart of the selection process of the included studies.

Accordingly, the present systematic review collected 27 research studies that specifically investigated the role of curcumin in reducing drug-induced toxic behaviors on male reproductive organs. Key findings include: i) In animal models of drug-induced infertility, curcumin administration showed significant improvements in sperm morphology, number, motility, and viability, ii) Testosterone levels for curcumin-treated animals were reduced with a decrease in MDA and nitric oxide (NO) and an increase in the antioxidant enzymes such as SOD and GPx in the testes, iii) Light microscopic evaluation showed that curcumin effectively reduced drug-induced changes in the shape of seminiferous tubules and the general morphology of the testes, iv) Curcumin suppressed drug-induced testicular cell apoptosis, which was accompanied by changes in the levels of apoptosis-related genes (e.g., Bcl-2 and bcl-2-like protein 4 (Bax)) and proteins, and v) Furthermore, curcumin demonstrated protection in pre-, co-, or post-drug treatment models, which may indicate a wide range of clinical applications (Figure 1). Below are the main drugs that induce reproductive toxicity in males and that are mitigated by curcumin.

#### 3.1.1 Bleomycin

In human testicular germ cell lines, such as NTera-2 and NCCIT, curcumin has demonstrated protective effects against bleomycin-induced oxidative stress; however, this protection may not always be advantageous from a clinical standpoint. Bleomycin’s pro-oxidative chemotherapeutic action may be countered by curcumin’s antioxidant qualities, which could lessen the drug’s effectiveness in treating cancer. Co-administration of curcumin dramatically reduced apoptosis in treated cells, as shown by Cort et al. (2013), indicating that concurrent use may compromise the intended cytotoxic effects of bleomycin (Cort et al., 2013). This finding highlights the intricate and context-dependent nature of curcumin’s action, particularly when combined with traditional chemotherapeutic agents. Although curcumin’s therapeutic benefits—especially its anti-inflammatory and antioxidant qualities—are widely acknowledged, in certain situations where oxidative stress is a mechanism of drug action, these benefits may work against the patient. Thus, the findings highlight the significance of timing, dosage, and context in the clinical application of curcumin rather than contradicting the review’s primary goal.

#### 3.1.2 Cisplatin

Cisplatin is one of the most common chemotherapy drugs for different types of cancer, but it harms the male endocrine system. It has been postulated that cisplatin causes reduced sperm motility, lower sperm concentration, and an increased percentage of abnormal sperm because of oxidative stress and testicular toxicity (Kaya et al., 2019; Shokri et al., 2020). Additionally, it disrupts hormonal balance by decreasing testosterone and follicle-stimulating and luteinizing hormones. The side effects of cisplatin are that it affects the spermatogenic cells and causes temporary or permanent sterility (Aksu et al., 2016; Moradi et al., 2024).

The results of the first study confirmed changes in the levels of the transcription factor nuclear factor kappa B (NF-κB)/p65, caspase-3, and 8-hydroxy-2' -deoxyguanosine (8-OHdG) in the germinal epithelium and Leydig cells of testicular animals after cisplatin administration. These markers are related to oxidative stress, inflammation, and apoptosis, some of which are underlying cisplatin-induced testicular dysfunction. Additionally, this study demonstrates that both curcumin and amifostine significantly mitigate the adverse effects of cisplatin on testicular tissue. These results provide evidence for the observed antioxidant, anti-inflammatory, and anti-apoptotic effects of curcumin, which are achieved through the reduction of NF-κB/p65, caspase-3, and 8-OHdG in renal cells under oxidative stress, inflammation, and apoptosis induced by cisplatin, respectively. The observed protective effects of curcumin may be attributed to its antioxidant, anti-inflammatory, and anti-apoptotic properties. Curcumin modulates distinct signaling pathways. NF-κB signaling is specifically involved in inflammation and apoptosis. In addition to the complications caused by free radicals and oxidative stress. Curcumin may also protect against cisplatin-induced testicular toxicity (Mercantepe et al., 2018).

The second study aimed to investigate the ability of curcumin to mitigate the effects of cisplatin-induced oxidative stress on rat testes. The present research demonstrates that curcumin is capable of attenuating cisplatin-induced oxidative stress, inflammation, and apoptosis. This compound also antagonizes the mitogen-activated protein kinase (MAPK) and NF-κB signaling pathways, exhibiting a protective effect on the testis. In this work, curcumin improved male reproductive health and histopathological changes. Based on the results of the present study, curcumin could be considered a potential adjuvant that contributes to gonadal preservation during cisplatin treatment (Ilbey et al., 2009).

Another study assessed the potential of curcumin to attenuate cisplatin-induced testicular toxicity in rats. According to the results of this work, curcumin antagonizes the androgen receptors and the Oct2 gene, thereby mitigating the adverse effects of cisplatin on the testicular parenchyma. Curcumin has additional activity in preserving testosterone concentrations and supporting spermatogenesis. These results suggest that curcumin may have therapeutic potential in addressing reproductive dysfunctions induced by chemotherapy (Fard et al., 2021).

#### 3.1.3 Cyclophosphamide

In another study, the author highlights the use of curcumin in protecting fertility. These findings suggest that curcumin supplementation leads to a restoration of hormonal levels, an increase in motility, and a reduction in abnormal sperm compared to those treated with cyclophosphamide (CPA) alone. Additionally, there was an improvement in testicular weight, indicating enhanced testicular health with curcumin supplementation. This study also demonstrates that curcumin’s protective ability can be attributed to factors such as its antioxidant properties and its capacity to modulate hormone levels. Altogether, these outcomes categorize curcumin as a potential therapeutic compound for mitigating the effects on fertility caused by CPA (Akomolafe and Aluko, 2020).

#### 3.1.4 Cyclosporine A

The immunosuppressive drug cyclosporine A (CsA) is a therapeutic agent used in organ transplantation and the treatment of autoimmune diseases. Although CsA shows benefits in the treatment of these medical disorders, CsA has been reported to produce a toxic effect on male genital organs. CsA therapy has been calculated to lower testosterone concentrations in the testes, raise gonadotropins, and hamper spermatogenesis. This translates into reduced fertility and infertility in animals. CsA is toxic to male genital function, and its toxicity can arise during treatment and has a dose-related manifestation, especially for patients under extended treatment regimens (Horoz et al., 2024).

Gazipoo et al. showed that curcumin affects the potential testicular damage when administered with CsA. It also acts as a free radical scavenger, reducing inflammation by controlling the levels of oxidative stress and apoptosis in CsA-induced rat testes. Curcumin action reduces CsA-induced testicular damage through modulation of the miR-34a/sirtuin 1(SIRT1) signaling pathway, which protects testicular tissue from any damage. This treatment results in a decrease in Nox4 levels and an increase in SIRT-1 levels, both of which are essential for safeguarding testicular tissue. In addition, the present results showed that curcumin administration also increased testicular weight and testicular coefficient, thus indicating its protective effect on testicular tissue. On the same note, curcumin is an antioxidant that protects organs against various diseases by modulating the formation of free radicals (Ghazipour et al., 2020).

#### 3.1.5 Dexamethasone

Dexamethasone (DEX) is a synthetic glucocorticoid that exerts a toxic effect on the male reproductive system, resulting in malformations and low sperm count. A systematic study indicates that DEX can lead to hypogonadism, low testosterone, decreased sperm production, and therefore low fertility (Khorsandi et al., 2013; Alahmar et al., 2019). In addition, sub-chronic toxicity has been known to cause histopathological changes in the testes, including testis atrophy and reduced sperm quality (Gür et al., 2005; Sahoo et al., 2008; Orazizadeh et al., 2010; Agarwal, 2011).

In another study, Khorsandi et al. observed that the expression of Bcl-2, an anti-apoptotic protein, was increased in the curcumin and DEX-treated group, which might be due to the modulatory effect of curcumin on DEX. However, DEX-induced abnormalities in spermatogenesis were observed, including epithelial vacuolation, germ cell destruction, a reduction in seminiferous tubule diameter, and a decrease in sperm head count, often indicative of maturation arrest. Furthermore, these changes induced by DEX were highly significant and significantly attenuated by curcumin + DEX, indicating a protective role of curcumin in testicular toxicity. The findings of this study showed that curcumin could prevent testicular toxicity in rats treated with DEX. This protection was likely achieved through the upregulation of the anti-apoptotic protein B-cell lymphoma 2 (Bcl-2) by curcumin, as well as the improvement of spermatogenic disorders induced by DEX. The above findings suggest that curcumin may have a potential therapeutic role in mitigating the adverse effects of DEX on testicular function (Khorsandi et al., 2013).

#### 3.1.6 Diclofenac sodium

Diclofenac sodium is a nonsteroidal anti-inflammatory drug (NSAID) used to treat body pain and inflammation. However, in recent years, some examinations have pointed out some side effects on the male reproductive system. Scientific studies have shown that diclofenac sodium has an inhibitory effect on reproductive organs, including the testis, epididymis, and prostate, as well as on sperm concentration, motility, and testicular function, in a dose-dependent manner. This histopathological examination of spermatogenic testicular tissue also revealed pathophysiologically significant changes, including the shrinkage of the seminiferous tubules and a reduction in the spermatogenic population, which may impact fertility. Altogether, these studies demonstrate that the use of diclofenac sodium affects the hormonal system and male reproductive organs. Thus, the drug should be taken with appropriate precautions (Vyas et al., 2019).

One study demonstrated that curcumin, coumarin, and honey may have protective effects on testicular function in adult mice exposed to diclofenac sodium. These natural products could be used as a useful preventive measure for testicular dysfunction. They operate these mechanisms, inhibition of cell death, and protection of cells against damage. These investigations demonstrate that curcumin, coumarin, and honey can further contribute to and attenuate diclofenac-induced testicular injuries by inhibiting lipid peroxidation and increasing the antioxidant activities of glutathione (GSH) and SOD levels, respectively. Ultimately, protection against diclofenac sodium-induced testicular dysfunction in adult mice is conferred by natural compounds such as these (El Mileegy et al., 2023).

#### 3.1.7 Doxorubicin

Doxorubicin (DOX) is a member of the anthracycline family of antibiotics derived from *Streptomyces* peucetius and is a potent agent for treating neoplastic diseases, thus having a broad application in cancer treatment. Nevertheless, its application has been linked to severe adverse effects, particularly reproductive toxicity. Reports from the past revealed that DOX induces testicular lesions in male animals through atrophy of the testes, diminution of the number of sperm, and morphological alterations of the sperm. It influences the hypothalamic-pituitary-gonadal (HPG) axis and harms testosterone and sperm, resulting in adverse effects on hormonal levels. Furthermore, oxidative stress and germ cell toxicity are risk factors for infertility, as is the need to protect germ cells during chemotherapy (Levi et al., 2015; Mohan et al., 2021; Zi et al., 2022).

Hikaze Aksu et al. also demonstrate the extensive improvements induced by curcumin on DOX-induced testicular toxicity in male rats. The data presented demonstrate that DOX treatment induces oxidative stress, impairs sperm motility, reduces the percentage of viable sperm cells, and increases MDA levels. It increases testicular necrosis and spermatogenic degeneration and causes DNA damage. These effects are consistent with previous studies that have shown the toxic effect of DOX on testicular tissues. According to the findings of the present study, it is suggested that the antioxidant effect of curcumin is mediated by reducing the free radical load and increasing cellular antioxidants. The reduced testicular toxicity, as demonstrated by curcumin treatment at different concentrations, supports the therapeutic role of curcumin in reducing the effects of DOX on male fertility in DOX-intoxicated rats. Based on the results, DOX is one of the most widely used chemotherapeutic agents. Still, the use of DOX has been limited due to toxic effects on the organism, including testicular toxicity. According to the findings of this study, curcumin could be helpful in adjuvant therapy to prevent DOX-associated long-term testicular toxicity and reduced fertility in cancer patients. Hence, further research is necessary to determine the optimal dose and timing of curcumin administration to achieve a constructive approach to mitigating DOX-induced testicular damage. Furthermore, the findings of this study can be generalized to other types of chemotherapy that affect gonadal function, potentially aiding in the development of new strategies to preserve fertility in men with cancer (Aksu et al., 2019).

#### 3.1.8 Irinotecan

Irinotecan (IR) is an anticancer and chemotherapeutic agent widely used in the treatment of cancer, especially colorectal cancer, and has toxic effects on male gonadal tissue. Uyanik et al. (2021) investigated the effects of IR on the testes, as it is a water-soluble derivative of the topoisomerase I group of antineoplastic drugs. IR has been used for a long time in cancer treatment, even though in some cases it has destructive effects on tissues such as the testis. The present work showed that; IR treatment significantly decreased GSH, SOD, GPx, and catalase (CAT) in rat testicular tissue. Curcumin treatment may have some potential in preventing this decrease in GSH, SOD, CAT, and GPx levels. In addition, histopathological observations revealed that the seminiferous tubules of IR-treated rats exhibited interstitial edema, vacuolization, and degeneration of tubular and ductal cells within the seminiferous tubules. In the IR + curcumin-treated group, curcumin reduced the histopathological changes induced by IR. In addition, IR treatment also increased abnormal sperm counts, but it was then observed that this effect could be reversed using curcumin treatment.

#### 3.1.9 Methotrexate

Methotrexate (MTX) is an immunosuppressive and cytotoxic medication used in the management of autoimmune diseases, including rheumatoid arthritis, psoriasis, and certain types of cancer. Many people use beta-blockers in the management of these diseases, but this should be done with caution because it affects the male reproductive system. These data suggest that while exposure to MTX has adverse effects on sperm quality in short-term studies, most experimental reports have not identified any long-term harmful effects of MTX on male fertility (Grosen et al., 2017; Perez-Garcia et al., 2022; 2023).

A study demonstrated that curcumin protects the testes of mice from MTX toxicity by suppressing the signaling proteins p38-MAPK and NF-κB. These data indicated that curcumin was effectively used to prevent MTX-induced testicular toxicity through these signal pathways. Curcumin may mitigate the effects of MTX, including body weight gain, morphological changes, and histological alterations. It also reduces the extent of direct contact of testicular tissue with MTX exposure toxicity by reducing the p-p38 MAPK immune response and NF-κB reactivity (Kilinc and Uz, 2021).

#### 3.1.10 Metronidazole

Metronidazole (MTZ) is an antibiotic used for different infections in patients with a toxic impact on the male reproductive system. Thus, high dosages of MTZ demonstrated fully reversible toxic effects on male fertility in rodents due to changes in the course of spermatogenesis and sperm characteristics. In particular, testicular and epididymal mass shrinkage, often associated with germ cell necrosis, has occurred especially at doses of 250 mg/kg and above, which would therefore suggest impaired reproductive capacity (McClain et al., 1989; El-Nahas and El-Ashmawy, 2004; Kumari and Singh, 2013).

One study has shown that the administration of MTZ diminishes secretion in the seminal vesicles of mice. In contrast, curcumin preserves the organs by maintaining gland integrity and promoting vesicular fluid secretion. MTZ did not alter structural parameters, except for secretion, which was disrupted by curcumin. This study also demonstrates how curcumin reduces the activity of MTZ on seminal vesicle secretion, a key factor in determining sperm stability and fertility. This is probably due to the protective properties of curcumin, which acts as an antioxidant and anti-inflammatory agent against the toxic effects of MTZ, particularly oxidative stress (Noorafshan and Karbalay-Doust, 2012).

Another investigation examines the effects of curcumin on the structural morphology of seminiferous tubules and Leydig cells in rats exposed to MTZ. The findings show that MTZ significantly reduces both the size and organization of the seminiferous tubules, as well as the number of spermatocytes and spermatids. However, the detrimental effects of MTZ on testicular parameters are reduced by curcumin administration when used in combination with MTZ. Stereological methods quantitatively assess these changes and highlight the role of curcumin as a protective agent against drug-induced reproductive toxicity. Overall, this study aligns with previous research that supports the protective effects of curcumin in mitigating the toxic effects of MTZ on the male reproductive system (Noorafshan et al., 2011).

In the study presented by Noorafshan et al., the protective potentials of curcumin on the seminiferous tubules and Leydig cells of mice treated with MTZ was analyzed. The stereological approaches used in this study indicate that curcumin enhances the drug-induced structural changes. In this way, curcumin helps restore the volume and density of these reproductive structures to nearly normal levels. This protection is considered derived from the antioxidant properties of curcumin. In conclusion, curcumin exhibits significant therapeutic potential in protecting the male reproductive system against the adverse effects of pharmaceutical agents (Noorafshan et al., 2011).

#### 3.1.11 Morphine

Morphine has been demonstrated to produce adverse effects on male fertility, which is mainly represented by sperm quality and number. Chronic use of morphine lowers levels of testosterone and impairs spermatogenesis activity, and subsequently men’s fertility (Gür et al., 2005; Roshankhah et al., 2017). However, morphine enhances the production of oxidative stress in the testes, decreasing sperm quality and the overall potential of reproduction (Ghasemi-Esmailabad et al., 2022).

Studies have shown that male reproductive health may counteract the noxious effects of morphine through the opposing application of curcumin. Sperm motility, sperm count, and testicular weight increased considerably after the administration of curcumin. It thus showed that curcumin can reduce the noxious effect of morphine on the production and quality of sperm. Curcumin incorporation also restored serum testosterone distribution and reduced morphine-mediated testicular histopathological changes, thereby confirming the protective role of curcumin in male gonadal tissue. All these findings have significant implications for the potential of curcumin as a therapeutic agent in opioid-related reproductive disorders (Roshankhah et al., 2017).

#### 3.1.12 Paclitaxel

In cancer therapy, paclitaxel is a cytotoxic agent used in the treatment of a variety of cancers, with particular emphasis on breast and ovarian cancer. This property is achieved by attaching to microtubules and preventing disruption of cell division by microtubule polymerization (Zhang et al., 2023).

The effect of curcumin on paclitaxel-mediated oxidative stress and induced DNA damage in the testis tissue of adult male rats has also been studied. It is also shown that testicular damage from paclitaxel can be reduced through a combination of paclitaxel and curcumin. The main reason for this is the improvement in histological parameters, increased testosterone levels, and reduced DNA damage in the curcumin + paclitaxel group compared to the paclitaxel-treated group. It is suggested that this protective effect of curcumin against the damaging effect of paclitaxel on male reproductive health is due to its antioxidant effect (Balcioğlu et al., 2023).

#### 3.1.13 Paracetamol

The administration of paracetamol has also been associated with undesirable effects on the male reproductive system concerning semen quality. Studies have shown that high doses of paracetamol can cause impaired sperm motility and viability, as well as changes in the morphology of spermatozoa, thereby impairing fertilization capacity (Smarr et al., 2017; Banihani, 2018). This can be explained by paracetamol’s ability to suppress testosterone synthesis and induce oxidative stress, with apoptotic effects on spermatocytes (Lee et al., 2023). Moreover, epidemiological evidence suggests that increased urinary levels of paracetamol are associated with lower sperm quality parameters, including DNA fragmentation and motility (Smarr et al., 2017; Lee et al., 2023).

A study has concluded that the use of paracetamol affects the male reproductive system and sperm quality detrimentally. It exerts its negative effects at these concentrations on sperm motility and viability and changes in sperm morphology that reduce the chances of fertilization; it can also affect testosterone levels and cause oxidative stress if taken for a long time, and is are leading factor in male infertility (Banihani, 2018).

El-Maddawy et al. also noted that curcumin has the potential to reduce the effects of paracetamol toxicity on serum testosterone, luteinizing hormone (LH), and follicle-stimulating hormone (FSH) compared to the control group. However, curcumin was able to help restore catalase and SOD activities, along with GSH levels, induced by paracetamol in the testes. In addition, after curcumin treatment, a reduction in the histopathological changes of the testes related to paracetamol-induced toxicity, as well as degenerative and necrotic changes, was observed. Curcumin also increased the number of sperm cells, sperm forward progression, and morphological abnormalities of the sperm affected by paracetamol (El-Maddawy and El-Sayed, 2018).

Also, in a study, the author investigated the role of curcumin in preventing paracetamol-induced testicular toxicity in adult male rats. Histological and histochemical analyses showed that curcumin has the potential to ameliorate paracetamol-induced degeneration of seminiferous tubules and reduce spermatogenesis. Histological characterization confirmed that curcumin reduced the levels of oxidative stress-induced damage and tissue apoptosis induced by paracetamol. In addition, general biochemical assays confirmed that curcumin improved antioxidant enzyme activities and reduced MDA levels, which is evidence of curcumin’s antioxidant activity. Consequently, this work emphasizes the ability of curcumin to protect against drug-induced reproductive toxicity in future generations (Mohammed and Sabry, 2020).

#### 3.1.14 Sertraline

Sertraline is a selective serotonin reuptake inhibitor (SSRI) and is primarily used to treat depression and anxiety disorders. Research shows that sertraline has several effects on men’s health and libido, erectile function, and sperm count. Several studies suggest that SSRIs such as sertraline can cause sexual dysfunction in the areas of low libido and erectile dysfunction, which may be due to changes in serotonin, which causes changes in neurotransmission in the penis (Altman, 2001).

Another report showed that curcumin may reduce the testicular toxicity induced by sertraline in rats by inhibiting oxidative stress. This protective role of curcumin is assumed due to the antioxidant property of this compound that may counteract the toxic effect of sertraline on testicular tissue. In this study, researchers also demonstrated that curcumin can reduce oxidative stress, enhance antioxidant enzymes, and mitigate the harmful effects of several chemicals on testicular tissue. Therefore, curcumin should be considered a potential therapeutic agent to protect the male reproductive system (Louei Monfared and Yousefizadeh, 2023).

#### 3.1.15 L-thyroxine (T4)

Some studies suggested that L-thyroxine (T4) influences the hypothalamic-pituitary-gonadal axis, controlling testosterone production and, consequently, spermatogenesis in the male reproductive system. Hypothyroidism adversely affects testosterone and sperm concentration, while in hyperthyroidism, sperm motility and morphology are influenced. However, it is agreed that treating hypothyroidism in men with T4 improves semen quality and testosterone and is therefore essential for the male reproductive system (Agarwal, 2011; Alahmar et al., 2019).

Another study also found that curcumin and vitamin C reduced lipid peroxide and protein carbonyl levels in the testes of mice treated with T4. In addition, both antioxidants increased SOD and CAT levels. This study shows that curcumin does not improve sperm count or sperm fertility quality, but it can do so if used with vitamin E. The combination of curcumin and vitamin E increases GSH. These changes are also accompanied by an increase in the GSSG/GSH ratio in mitochondrial compartments. This study also demonstrates the beneficial effect of curcumin and vitamin E in reducing oxidative stress induced by T4 in the testes of mice. While both antioxidants have the same efficiency in replenishing antioxidant enzymes, vitamin E seems to have better effects on sperm quality. Protection with the combination of curcumin and vitamin E suggests that the combination of antioxidants may provide better protection against oxidative stress in hyperthyroid conditions (Sahoo et al., 2008).

### 3.2 The protective effect of curcumin on the male reproductive system in humans

Curcumin, the main active compound of *Curcuma longa* L [Zingiberaceae], has been widely studied for its antioxidant and anti-inflammatory properties, which are particularly relevant in the context of male infertility where oxidative stress plays a significant role in sperm dysfunction and DNA damage (Agarwal et al., 2014). The role of curcumin has been pharmacologically reviewed in relation to the male reproductive system, with a focus on specific aspects of sperm functionality and sensitivity to oxidative stress. Karakus et al. conducted research into how curcumin affects the parameters of cryopreserved sperm samples, therefore inferring from its antioxidant properties that curcumin has an enhancing effect on sperm morphology and enhanced stability of the plasma membrane. This study underlined the negative impacts of cryopreservation on sperm quality. Overall, the results suggested that curcumin could have a positive effect on the post-thaw features of sperm (Nur Karakus et al., 2021).

Another study examines the use of curcumin to improve cryo-damage in the context of preserved spermatozoa. Results show that curcumin increased the progressive motility, viability, and DNA compactness of sperm after the freeze-thaw cycle. In this study, this is attributed to the antioxidant activity of curcumin, which significantly lowers oxidative stress. Thus, the inclusion of curcumin into the current cryopreservation procedures enhances fertility success rates. It further extends to explore the various possibilities of curcumin application as a protective agent in assisted reproductive technology (Santonastaso et al., 2021).

A review study discusses the effects of curcumin on asthenozoospermia, an andrological disorder characterized by reduced sperm motility. Results indicated that curcumin enhanced the reduction of ROS, which hurts sperm quality. Additionally, curcumin reduced the level of MDA, a marker for oxidative stress. This study reveals that curcumin acts *via* nuclear translocation of the nuclear factor erythroid 2-related factor 2 (NRF2) protein, leading to increased transcription of antioxidant genes. A critical cellular defense against oxidative damage is thus provided. In brief, it appears that curcumin is a promising therapeutic agent for treating male infertility mediated by oxidative stress and may also serve as a potential treatment strategy for asthenozoospermia (Zhou et al., 2020).

**FIGURE 2:**
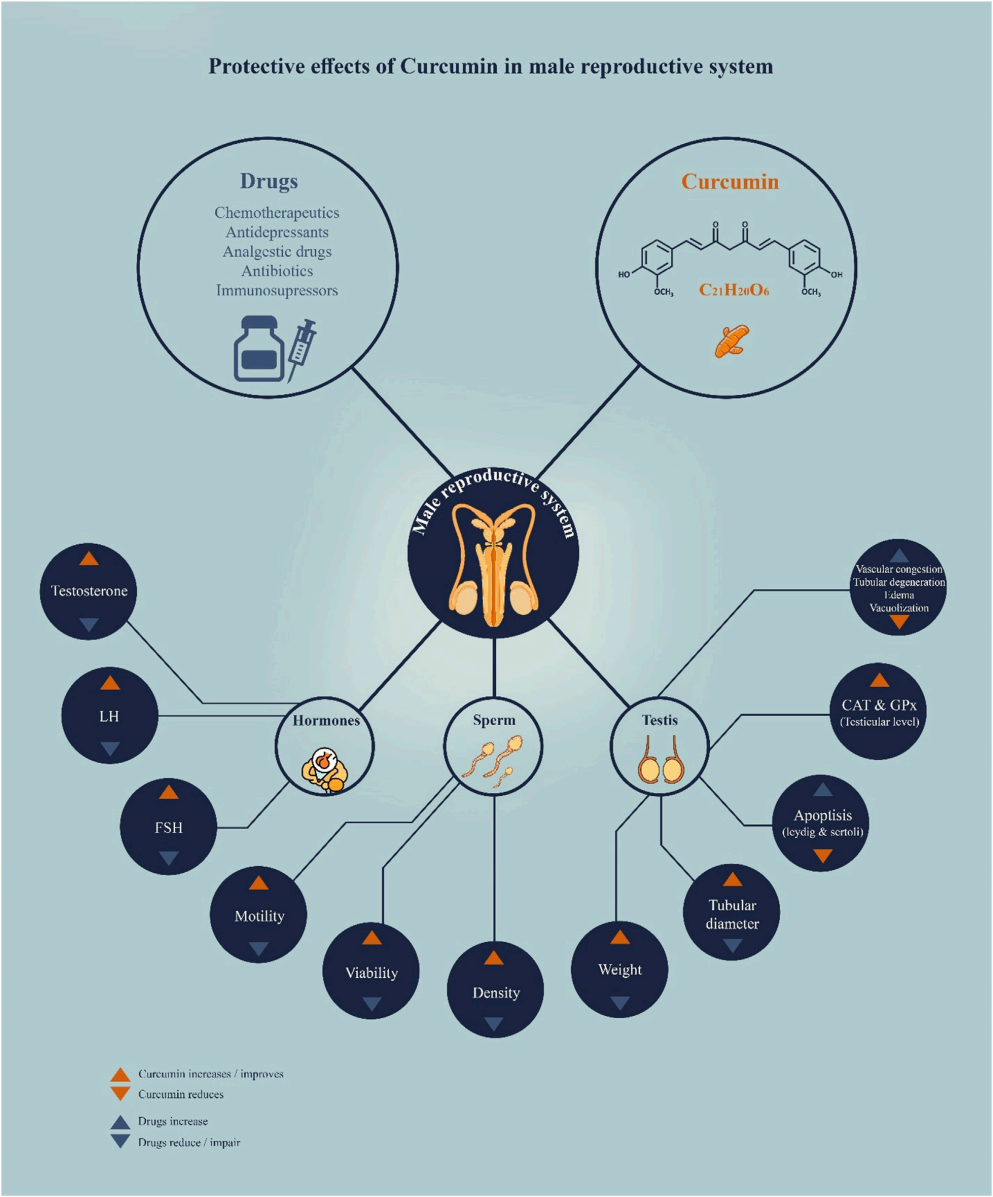
Protective effect of curcumin on the male reproductive system.

A clinical trial also evaluated the effects of curcumin nano-micelles on male infertility in a randomized group of sixty infertile men. Individuals treated with 80 mg/day of curcumin showed a marked increase in total sperm count, sperm concentration, and sperm motility. With curcumin treatment, markers related to oxidative stress and inflammatory indices were reduced. There was an improvement in reproductive hormones. These results not only demonstrate the clinical benefits of curcumin in treating male infertility but also highlight the advantages of nanotechnology in enhancing the bioavailability and therapeutic efficacy of curcumin. Results obtained in this work confirm that nano-micellar curcumin may have positive effects on male fertility; therefore, further research in this field is necessary (Alizadeh et al., 2018). Consequently, the justification for using curcumin lies in its target mechanism, which includes the modulation of several diseases, reduction of inflammation, regulation of apoptosis, and structural recovery. Such multiple functions make it a promising agent in the management of male infertility, especially in cases caused by oxidative and idiopathic stress (Figure 2).

### 3.3 Mechanisms responsible for the protective effects of curcumin against toxicity induced by drugs

**FIGURE 3:**
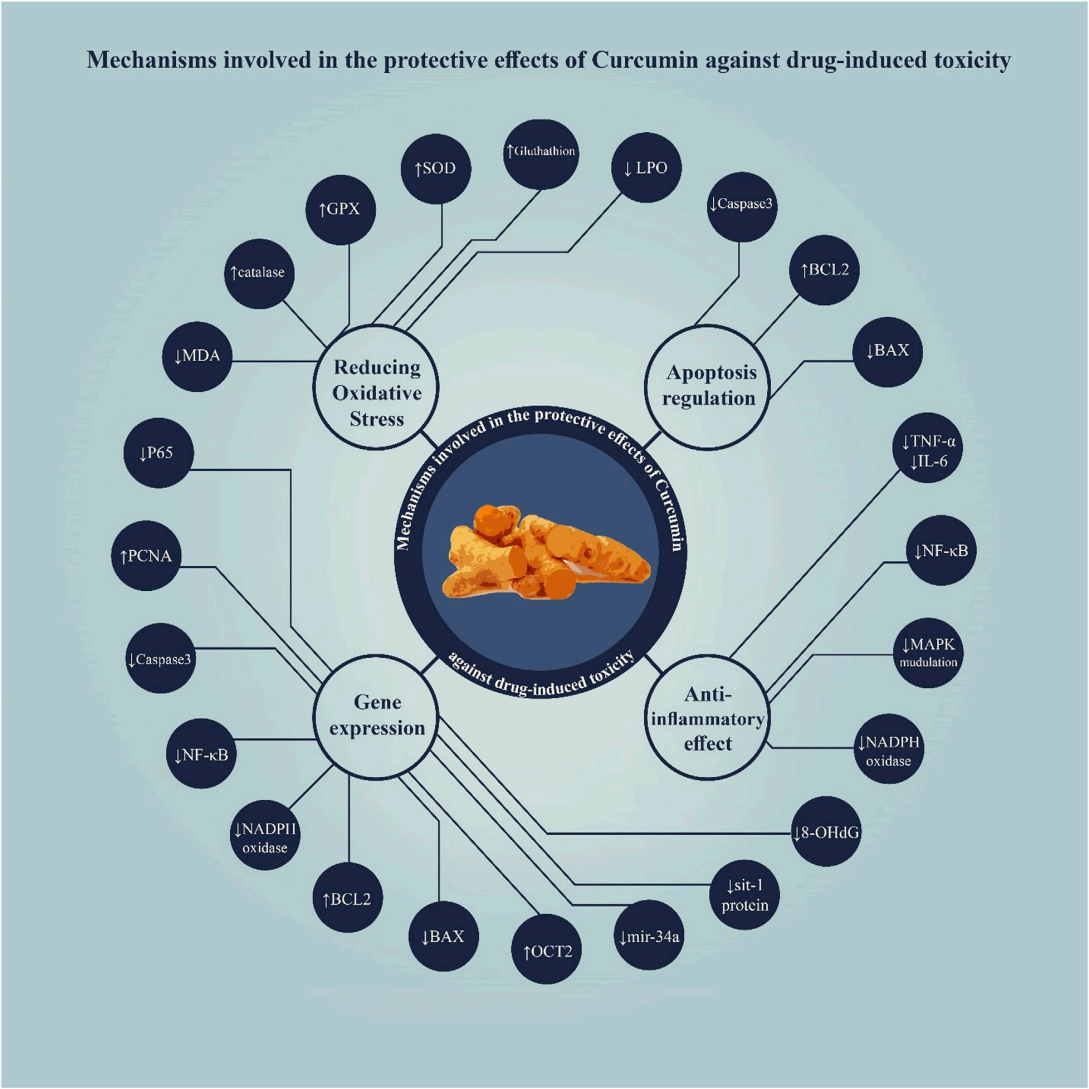
Mechanisms underlying the protective effects of curcumin against drug-induced toxicity.

According to the results of the studies reviewed in this study, curcumin exhibits protective effects against drug-induced toxicity in the male reproductive system through several mechanisms, primarily including antioxidant properties and modulation of apoptosis pathways (Figure 3).

#### 3.3.1 Antioxidant activity

Curcumin is known for its potent antioxidant properties. It scavenges free radicals and ROS, which are often generated during drug metabolism and can lead to oxidative stress in reproductive tissues. By reducing oxidative stress, curcumin helps protect sperm cells and other reproductive tissues from damage. Additionally, curcumin enhances the activity of antioxidant enzymes, such as SOD and catalase, while simultaneously decreasing lipid peroxidation levels in testicular tissues, thereby preserving cell integrity and function (Rasool et al., 2024). Moreover, curcumin enhances the levels of GSH, a critical intracellular antioxidant that helps neutralize ROS. Increased GSH levels reduce oxidative stress and protect spermatozoa from damage (Dai et al., 2022).

Curcumin also significantly decreases lipid peroxidation levels, which is a marker of oxidative damage. By inhibiting lipid peroxidation (LPO), curcumin protects cell membranes in reproductive tissues from oxidative damage caused by drugs. MDA is a byproduct of lipid peroxidation and serves as an indicator of oxidative stress. Curcumin treatment has been associated with reduced MDA levels, indicating decreased lipid peroxidation and improved cellular health (Flora, 2011; Rasool et al., 2024).

This compound also enhances the activity of antioxidant enzymes, including GPX and CAT, further. These enzymes contribute to the metabolism of hydrogen peroxide and toxic substances, thereby strengthening the antioxidant defense system in reproductive tissues (Zhang et al., 2018; Afshari et al., 2023).

#### 3.3.2 Anti-inflammatory effects

Drug-induced toxicity is frequently associated with inflammatory responses, which in turn may lead to accelerated tissue damage. Curcumin is a regulator of inflammatory pathways by antagonizing pro-inflammatory cytokines and enzymes. Curcumin blocks the action of the NF-κB pathway, which leads to the transcription of various inflammatory mediators. This inhibition decreases the expression of the cytokines TNF-α and interleukin 6 (IL-6), and as a result, inflammation is controlled in the male reproductive system (Zhang et al., 2018).

#### 3.3.3 NADPH oxidase

Curcumin was found to be a nicotinamide adenine dinucleotide phosphate oxidase (NADPH) oxidase inhibitor responsible for the generation of ROS during inflammation. Curcumin, by inhibiting ROS production, mediates anti-inflammatory effects and thereby diminishes tissue-destructive inflammation in the male reproductive system (Oria et al., 2024).

#### 3.3.4 MAPK signaling pathway

Curcumin affected the MAPK signaling pathway, especially the extracellular signal-regulated kinase (ERK) pathway. This modulation controls cell-death responses to stress and survival signaling pathways in germ cells (Fang et al., 2018).

#### 3.3.5 Regulation of apoptosis

Curcumin modulates apoptotic proteins in response to drug-induced stress. It may also reduce apoptosis (programmed cell death) in germ cells and Leydig cells, which are crucial for male reproduction. (Fujiwara et al., 2021). This compound modulates the expression of Bcl-2 family proteins, increasing anti-apoptotic factors (e.g., Bcl-2) while decreasing pro-apoptotic factors (e.g., Bax). This balance helps maintain cell survival under toxic conditions (Tan et al., 2006; Wu et al., 2013).

#### 3.3.6 Caspase-3 activation

Curcumin may suppress the activation of caspase-3, which is a potent promoter of apoptosis in cortical neurons. In situations where toxicities occur, curcumin aids in maintaining fertility by moderating apoptosis in germ cells as well as Leydig cells (Meesarapee et al., 2014; Wang et al., 2020).

#### 3.3.7 Hormonal regulation

Curcumin is known to affect hormonal balance and plays a significant role in the male reproductive system. It increases testosterone levels and may have beneficial effects on the endocrine system. Leydig cells are hormone-producing cells that regulate testosterone synthesis, spermatogenesis, and overall male reproductive health. Curcumin enhances the activity of the steroidogenic enzymes in these cells (Ungurianu et al., 2022).

#### 3.3.8 DNA protection

Curcumin also has some effect in preventing DNA damage mediated by toxic drugs. This is important because DNA damage is fatal for spermatogenesis and for male fertility in general. Curcumin can prevent DNA damage by up-regulating the levels of DNA repair enzymes and down-regulating oxidative DNA adducts. This action ensures that genetic material is preserved in sperm cells (Balcioğlu et al., 2023).

#### 3.3.9 Modulation of signal transduction pathways

Curcumin interacts with various signaling pathways that are involved in cellular stress responses, including the MAPK pathway. By modulating MAPK signaling, curcumin can influence cell survival and proliferation signals, thereby enhancing cellular resilience against toxic insults (Ungurianu et al., 2022).

#### 3.3.10 Gene expression modulation

Curcumin influences the expression of several genes associated with oxidative stress and cellular repair. The downregulated miR-34a, which is also associated with apoptosis promotion, is regulated by curcumin. The gene SRT1 is related to the stress response of cells, and curcumin increases SIRT1 expression, thus improving cell viability (Heshmati et al., 2020). Likewise, proliferating cell nuclear antigen (PCNA) is required for DNA replication and it has been reported that curcumin increases the expression of PCNA to enhance cell division (Raoofi et al., 2021). Additionally, 8-OHdG is indicative of oxidative DNA damage, and it has been reported that curcumin downregulates its expression, suggesting an improvement in DNA protection. On the other hand, curcumin can also reduce the protein carbonyl levels, showing the extent of protein oxidation (Damiano et al., 2022). Similarly, curcumin treatment decreased thiobarbituric acid reactive substances (TBARS) levels, which establishes protection against lipid peroxidation (Ghasemi et al., 2019). Finally, curcumin may also modulate the organic cation transporter two gene (OCT2), which encodes proteins involved in the transport and metabolism of drugs, thereby altering the toxicity profiles of drugs (Medina-Pizaño et al., 2022).

The summary of the protective effects of curcumin against drug toxicity on the male reproductive system is shown in Table 1.

**TABLE 1:** Protective effects of curcumin against drug toxicity on male reproductive system.

Drug toxicity	Pharmacokinetic model	Treatment (dose and duration)	Outcome	References
Bleomycin	human malignant testicular germ cells	5 μM; 72 h	↓ Activities of caspases ↓ Bax ↓ Cyt-c ↓ LPO ↓ Protein carbonyls ↓ TBARS ↑ GSH	Cort et al. (2013)
Cisplatin	rat	10 mg/kg; 10 days	↑ Testosterone ↑ Testis AR gene expression ↑ OCT2 gene expression	Fard et al. (2021)
Cisplatin	Sprague Dawley rats	100 mg/kg/day; 7 days	↓ Expression of NF-κB/p65 ↓ Caspase-3	Mercantepe et al. (2018)
Cisplatin	male Wistar albino rats	200 mg/kg/day; 10 days	↑ Testosterone ↑ GSH ↑ GSH-Px ↓ MDA ↓ NO ↓ Seminiferous epithelial layers ↓ Significant maturation arrest ↓ Perivascular fibrosis Prevents the activation of caspase-3 Affects the MAPK and NF-κB signaling pathway	Ilbey et al. (2009)
Cyclophosphamide	Rat	20 mg/kg; 14 days	↑ FSH ↑ Prolactin ↑ Sperm motility ↑ Testicular weight ↓ Oxidative stress markers associated with CPA exposure Modulating the hypothalamic-pituitary-gonadal axis to restore hormonal balance	Akomolafe and Aluko (2020)
Cyclosporine A	Rat	40 mg/kg; 28 days	↑ Testis weight ↓ 8-OHdg ↓ Nox4 protein ↓ Bax ↓ Bcl-2 ↓ mir-34a ↓ sirt-1 protein	Ghazipour et al. (2020)
Dexamethasone	adult male NMRI	200 mg/kg/day; 10 days	↑ Bcl-2 protein expression, prevent epithelial vacuolizations, sloughing of germ cells ↓ Seminiferous tubule diameter ↓ Sperm heads	Khorsandi et al. (2013)
Diclofenac	mice	100 mg/kg; 21 days	↑ LH ↑ FSH ↓ Testicular LPO level ↑ GSH level, typical ST, spermatogenic cells, and interstitial cells of Leydig and Sertoli cells ↑ GR ↑ SOD2	(El Mileegy et al., 2023)
Doxorubicin	rat	30 mg/kg; 21 days	↑ Testicular weight ↓MDA Negative caspase-3 immunoreactivity ↓ NF-κB ↓ IL-1β ↓ IL-6 ↑ MMPs	Huyut et al. (2021)
Doxorubicin	rat	100 amd 200 mg/kg; 7 days	↑ Sperm motility rate ↑ Live sperm percentages ↑ Cellular antioxidants ↓ MDA levels ↓ Necrosis degeneration ↓ Slimming in the seminiferous tubules ↓ DNA damage in testes by inducing oxidative stress	Aksu et al. (2019)
Irinotecan	Rat	100 mg/kg/day, 1 month	↑ GSH ↑ SOD ↑ GPx ↑ CAT ↑ Vascular congestion, tubular degeneration, edema, vacuolization, and luminised cells in the seminiferous tubule ↓ TBARS ↑ Sperm motility and concentration	(Uyanik et al., 2021)
Methotrexate	rat	100 mg/kg/day; 14 days	↑ Seminiferous tubule diameter ↑ Germinal epithelium height ↑ Spermatogenesis ↓ Percentages of damaged seminiferous tubules ↓ Number of p-p38 MAPK immunopositive cells ↓ NF-κB immunoreactivity	(Kilinc and Uz, 2021)
Metronidazole	thirty balb/c mice	100 mg/kg; 14 days	↑ Gland and vesicular fluid volume	Noorafshan and Karbalay-Doust (2012)
Metronidazole	48 Balb/c mice	100 mg/kg; 30 days	↑ Volume ↑ Length ↑ Diameter of seminiferous tubules ↑ Height of the germinal epithelium Mitigate an increase in the number of Leydig cells	Noorafshan et al. (2011)
Metronidazole	mice	100 mg/kg; 16 days	Sperm count, motility, and tail length decreased to a lesser extent compared to mice treated with MTZ alone The reduction was mitigated in mice that received curcumin along with MTZ	(Karbalay-Doust and Noorafshan, 2011)
Metronidazole	Balb-c mice	100 mg/kgbw; 30 days	Protect spermatocytes ↑ Testis weight ↑ Testis volume ↑ Total epithelial volume ↑ Number of spermatids ↓ Germinal epithelium volume	Noorafshan et al. (2011)
Morphine	adult male rats	20–60 mg/kg; 28 days	↑ Sperm motility ↑ Sperm count ↑ Testis weight and testosterone levels Ameliorate the histopathological changes in the testis caused by morphine treatment	Roshankhah et al. (2017)
Paclitaxel	rat	15 mg/kg; 1, 7, 14, and 21 days of expriment	↓ Histological damage in the testicular tissue ↑ Testosterone levels ↓ DNA damage ↓ Number of apoptotic cells in the seminiferous tubules	Balcioğlu et al. (2023)
Paracetamol	rat	50 mg\kg; 10 days	↓ Loss of normal architecture of testicular tissue ↓ Wide interstitial spaces ↓ Loss of stratal arrangement of germinal epithelium with intercellular spacing	Mohammed and Sabry (2020)
Paracetamol	rats	200 mg/kg; 2 weeks	↑ CAT ↑ SOD ↓ LH ↓ FSH ↑ the paracetamol-induced degenerative changes and necrosis in the testes	El-Maddawy and El-Sayed (2018)
Sertraline	Wistar rats	100 mg/kg; 42 days	Prevented the decline in the testicular weights ↑ Tubular diameter ↑ Number of Leydig and Sertoli cell Normal histology of the germinal cells in the teste ↓ MDA ↑ Testicular levels of CAT and GPx	Louei Monfared and Yousefizadeh (2023)
L-thyroxine (T4)-induced hyperthyroid rats	Wistar male rats	30 mg/kg; 30 days	↓ LPO ↓ Protein carbonyl contents ↑ SOD ↑ CAT ↑ GSH/GSSG ratio in the mitochondrial fraction (MF) of T4-treated rats	Sahoo et al. (2008)

### 3.4 Protective effects of nanoformulation of curcumin against drug toxicity on the male reproductive system

Several studies have reported the protective effects of curcumin nanoformulations against drug-induced toxicity in the male reproductive system. These findings are summarized in Table 2.

**TABLE 2:** Protective effects of nanoformulation of curcumin against drug toxicity on the male reproductive system.

Nanoformulation	Drug toxicity	Pharmacokinetic model	Treatment (doses and duration)	Outcome	References
Nanonencapsulated curcumin (NEC)	Boldenone (BOL) (Equigan)	rat	once daily; 60 days	↓ MDA ↓ NO ↑ SOD ↑ GR ↑ Serum testosterone ↑ Semen quantity ↑ Sperm count ↑ Motility	Aly et al. (2021)
curcumin nanocrystals (NC)	Cyclophosphamide (CP)	male Swiss albino mice	4 mg/kg r 35 days; 35 days	↑ Sperm functional competence, sperm chromatin condensation, and seminiferous tubule architecture ↓ Apoptosis in testicular cells, ↓ LPO ↓ GSH ↑ Gpx4 ↑ CAT ↑ Ki67 positive expression ↑ Oct4	Poojary et al. (2021)
curcumin-loaded superparamagnetic iron oxide (Fe_3_O_4_) nanoparticles (NPs)	Methylphenidate (MPH)	Rat	5.4 mg/100 g; 21 days	↑ Sperm quality, certain cellular variables: round spermatid and Leydig cells ↑ Serum testosterone levels ↑ Sperm count ↑ Sperm motility ↓ Tlr-2 ↓ TNF-α ↓ IL6 ↑ PCNA ↑ Stra8	Raoofi et al. (2021)

#### 3.4.1 Boldenone

Boldenone (BOL) is an anabolic steroid that is active in veterinary practice as well as among bodybuilders for muscle-boosting purposes, and it is especially popular in the form of boldenone undecylenate. Some of the effects reported in the study include a reduction in seminal volume, sperm concentration, and motility, as well as a low serum testosterone level. After the use of this substance on male rabbits, a reduction of the testicular and epididymal mass was observed, which implies that this substance might cause testicular atrophy. Microscopical changes were, however, investigated extensively in the reproductive organs, and they deduced that the drug causes infertility in the long run. Therefore, the administration of BOL is very hazardous to male reproductive organs; hence, it should be undertaken with a lot of caution (Oda and El-Ashmawy, 2012; Bueno et al., 2017).

One study was conducted to evaluate the potential of vanonencapsulated curcumin (NEC) in preventing testicular injury and oxidative stress in male albino rats induced by boldenone exposure. In this research, the efficacy of NEC in mitigating testicular toxicity and oxidative stress associated with BOL treatment was assessed. The results suggested that simultaneous treatment with NEC and BOL minimized the adverse effects, including alterations in the morphological structure of seminiferous tubules, a decline in the population of spermatogonia, decreased testosterone levels, and elevated MDA levels. NEC exhibited an enhanced antioxidant potential and contributed to the reduction of cellular injury induced by BOL in the testes (Aly et al., 2021).

#### 3.4.2 Cyclophosphamide

Cyclophosphamide (CP) is an alkylating agent that was developed over 3 decades ago for the treatment of various forms of cancer. However, it also has carcinogenic effects on the male reproductive system primarily due to the cytotoxicity of the compound. CP inhibits the germ cells, Leydig cells, and Sertoli cells in the testis, resulting in decreased sperm count and testosterone levels (Ghobadi et al., 2017; Olowe et al., 2024). This drug induces toxic oxidative stress and damages the hypothalamic-pituitary-gonadal axis, which is essential for male fertility function (Song et al., 2023; Olowe et al., 2024). Therefore, the male receiving the CP treatment will be at risk of low fertility, besides other detrimental effects on the male genital tract (Ghobadi et al., 2017; Watcho et al., 2019).

The result of the experiment demonstrated that curcumin nanocrystals (NC) can reverse and prevent CP-induced testicular toxicity in mice. Adult mice receiving NC treatment had enhanced markers of sperm functional competence, increased sperm chromatin density, better spermatogenic tubule morphology, and decreased testicular cell apoptosis. Moreover, the present study demonstrated that NC administration altered the gene expression profiles of proliferation and pluripotency, as well as DNA damage and DNA repair, in the spermatogonial cells of prepubescent mice. In summary, the findings have indicated that NC may have the potential for regular use as a chemotherapy drug with potential use in male fertility preservation (Poojary et al., 2021).

#### 3.4.3 Methylphenidate

The effect of methylphenidate (MPH) in the treatment of attention-deficit/hyperactivity disorder (ADHD) has been investigated on the male reproductive system. One study concluded that MPH did not affect the morphology of sperm but had a favorable effect on the concentration and motility of sperm, both among current users and past users, compared to nonusers (Rezayat et al., 2020; Shalev et al., 2021). However, there are side effects with long-term use of MPH, which are: delayed puberty and hormonal changes. For instance, there was a case where a young man who used MPH developed testicular failure. Thus, in animals, high doses of MPH have been shown to reduce testicular weight and impair spermatogenesis (Rezayat et al., 2020; De Serrano et al., 2021). Consequently, the link between the MPH and male reproductive health is relatively inconclusive, and further studies are needed to establish the long-term impact of the substance.

The effects of curcumin nanoparticles on testicular tissue after the treatment of methylphenidate in rats were also studied in scholarly articles. In conducting this research, male rats were used, and the rats were divided into various groups. The first group received methylphenidate, and the second group received a curcumin nanosphere blended with methylphenidate. The outcomes reveal that curcumin nanoparticles improved sperm, stereological parameters, and testosterone more than the group receiving only MPH. Moreover, it was established that curcumin nanoparticles minimized the degree of testicular damage and apoptosis brought about by MPH, suggesting that curcumin nanoparticles could contain potential curative properties against the effects of MPH on testicular tissue (Raoofi et al., 2021).

## 4 Conclusion

Based on the reviewed studies, curcumin is demonstrated to relieve several drugs that induce male reproductive toxicity. According to the above research, the main responsible properties of curcumin include antioxidant, anti-inflammatory, and apoptosis pathway-inducing activities, all of which play a key role in enhancing the structural and functional integrity of the testes. Curcumin can prevent excessive or aberrant apoptosis in testicular cells induced by toxic drugs, thereby preserving testicular structure and spermatogenesis. The histological results of testicular architecture by curcumin show its potential usefulness in maintaining the dynamic environment provided by the seminiferous tubules and supporting cells, which are crucial for spermatogenesis. The modulation of apoptotic genes and proteins induced by curcumin suggests a potentially mediating role for programmed cell death, which is also disrupted in toxicant-induced testicular injury. These works demonstrating the positive role of curcumin in various therapeutic schedules (preventive, during, or after drug administration) make this molecule a highly adaptable one that can be used flexibly.

Curcumin and nano-curcumin show different protective effects against drug-induced toxicity in male reproduction. Because of the nano curcumin small-emulsion, nano curcumin bioavailability has been dramatically increased over regular curcumin, resulting in better absorption and metabolism in the body (Ahmed-Farid et al., 2017; Koohpeyma et al., 2024). Also, Lower doses of nano curcumin (e.g., 5 mg/kg) can provide protective effects similar to or better than the high doses of curcumin (e.g., 50 mg/kg) (Flora et al., 2013).

Nano-curcumin has been demonstrated to better promote sperm motility and overall spermatogenesis likely through its enhanced delivery and solubilizing properties (Draganski et al., 2018).

Nano-curcumin exhibits higher effectiveness in reducing oxidative stress markers and enhancing testicular tissue damage and repair as compared to conventional curcumin preparations. In addition, both formats are effective in increasing testosterone levels; however, nano-curcumin may produce more significant effects on hormonal balance and reproductive function (Koohpeyma et al., 2024).

Nano-curcumin has been the subject of an increasing amount of research as a more effective way to mitigate drug-induced male reproductive toxicity than traditional curcumin formulations. Nanocurcumin’s enhanced solubility, reduced particle size, and larger surface area result in a much higher bioavailability, which improves absorption and systemic distribution (Ahmed-Farid et al., 2017).

Curcumin nano-emulsion (CUR-NE) enhances sperm count, motility, morphology, DNA integrity, and testosterone levels significantly while lowering oxidative stress markers and restoring testicular structure, according to therapeutic studies conducted using cyclophosphamide-induced toxicity models (Ahmed-Farid et al., 2017; Poojary et al., 2021). Similarly, in mouse models of multiple sclerosis, curcumin nanomicelle enhanced spermatogenesis metrics, antioxidant capacity, and levels of LH, FSH, and testosterone, with histological confirmation of protective effects (Koohpeyma et al., 2024).

Therapeutic studies using cyclophosphamide-induced toxicity models have demonstrated that CUR-NE significantly improves sperm count, motility, morphology, DNA integrity, and testosterone levels, while restoring testicular structure and reducing oxidative stress markers (Ahmed-Farid et al., 2017; Poojary et al., 2021). Curcumin nanomicelle also improved spermatogenesis metrics, antioxidant capacity, and levels of LH, FSH, and testosterone in mouse models of multiple sclerosis, with histological confirmation of protective effects (Koohpeyma et al., 2024).

The reviewed studies employed various animal models, types of drug treatments, and curcumin doses, which should help explain the differences in the magnitude of the effects. The extrapolation of these studies into human subjects needs more clinical studies to prove the effectiveness and tolerance of curcumin in preventing drug-induced male reproductive toxicities. Further investigations should be directed toward understanding the specific molecular pathways in which curcumin offers its protection and the combined effectiveness of the antioxidant along with other agents. In conclusion, the systematic review provides strong evidence for the active part of curcumin in the prevention and attenuation of the effects of drug-induced toxicity on the male reproductive system. It may offer therapeutic possibilities for drug-induced reduced fertility in men.

## Data Availability

The original contributions presented in the study are included in the article/supplementary material, further inquiries can be directed to the corresponding authors.

## Glossary

ADHD: attention-deficit/hyperactivity disorder
Bax: bcl-2-like protein 4
Bcl-2: b-cell lymphoma 2
BOL: boldenone
CAT: catalase
CP: cyclophosphamide
CPA: cyclophosphamide
CRP: C-reactive protein
CsA: cyclosporine A
CUR-NE: curcumin nano-emulsion
DEX: dexamethasone
DNA: deoxyribonucleic acid
DOX: Doxorubicin
ERK: extracellular signal-irinotecanregulated kinase
FSH: follicle-stimulating hormone
GPx: glutathione peroxidase
GR: glutathione reductase
GSH: glutathione
GSSG: oxidized glutathione
HPG: hypothalamic-pituitary-gonadal
IL-6: interleukin 6
IR: irinotecan
LH: luteinizing hormone
MAPK: mitogen-activated protein kinase
MDA: malondialdehyde
MPH: methylphenidate
MTX: methotrexate
MTZ: metronidazole
NADPH: nicotinamide adenine dinucleotide phosphate oxidase
NC: curcumin nanocrystals
NCCIT: human embryonic carcinoma
NEC: nanonencapsulated curcumin
NF-κB: transcription factor nuclear factor kappa B
NO: nitric oxide
NRF2: nuclear factor erythroid 2-related factor 2
NSAID: nonsteroidal anti-inflammatory drugs
NTera-2: human teratocarcinoma
8-OHdG: 8-hydroxy-2' –deoxyguanosine
PCNA: proliferating cell nuclear antigen
RNA: ribonucleic acid
ROS: reactive oxygen species
SIRT1: sirtuin 1
SOD: superoxide dismutase
SSRI: selective serotonin reuptake inhibitor
T4: L-thyroxine
TBARS: thiobarbituric acid reactive species
TNF-α: tumor necrosis factor alpha.
